# Mucus-penetrating and permeation enhancer albumin-based nanoparticles for oral delivery of macromolecules: Application to bevacizumab

**DOI:** 10.1007/s13346-023-01454-0

**Published:** 2023-10-26

**Authors:** Cristina Pangua, Socorro Espuelas, María Cristina Martínez-Ohárriz, José Luis Vizmanos, Juan M. Irache

**Affiliations:** 1 NANO-VAC Research Group, Department of Pharmaceutical Sciences, School of Pharmacy and Nutrition, University of Navarra, 31008 Pamplona, Spain; 2 Department of Chemistry, University of Navarra, 31008 Pamplona, Spain; 3 Department of Biochemistry & Genetics, School of Sciences, University of Navarra, 31008 Pamplona, Spain; 4 Institute for Health Research (IdiSNA), 31008 Pamplona, Spain

**Keywords:** Permeation enhancer, Nanoparticles, Bevacizumab, Mucus-permeating, Bioavailability, Sodium deoxycholate

## Abstract

The oral administration of therapeutic proteins copes with important challenges (mainly degradation and poor absorption) making their potential therapeutic application extremely difficult. The aim of this study was to design and evaluate the potential of the combination between mucus-permeating nanoparticles and permeation enhancers as a carrier for the oral delivery of the monoclonal antibody bevacizumab, used as a model of therapeutic protein. For this purpose, bevacizumab was encapsulated in PEG-coated albumin nanoparticles as a hydrophobic ion-pairing complex with either sodium deoxycholate (DS) or sodium docusate (DOCU). In both cases, complex formation efficiencies close to 90% were found. The incorporation of either DS or DOCU in PEG-coated nanoparticles significantly increased their mean size, particularly when DOCU was used. Moreover, the diffusion in mucus of DOCU-loaded nanoparticles was significantly reduced, compared with DS ones. In a *C. elegans* model, DS or DOCU (free or nanoencapsulated) disrupted the intestinal epithelial integrity, but the overall survival of the worms was not affected. In rats, the relative oral bioavailability of bevacizumab incorporated in PEG-coated nanoparticles as a complex with DS (B-DS-NP-P) was 3.7%, a 1000-fold increase compared to free bevacizumab encapsulated in nanoparticles (B-NP-P). This important effect of DS may be explained not only by its capability to transiently disrupt tight junctions but also to their ability to increase the fluidity of membranes and to inhibit cytosolic and brush border enzymes. In summary, the current strategy may be useful to allow the therapeutic use of orally administered proteins, including monoclonal antibodies.

**Graphical Abstract:**

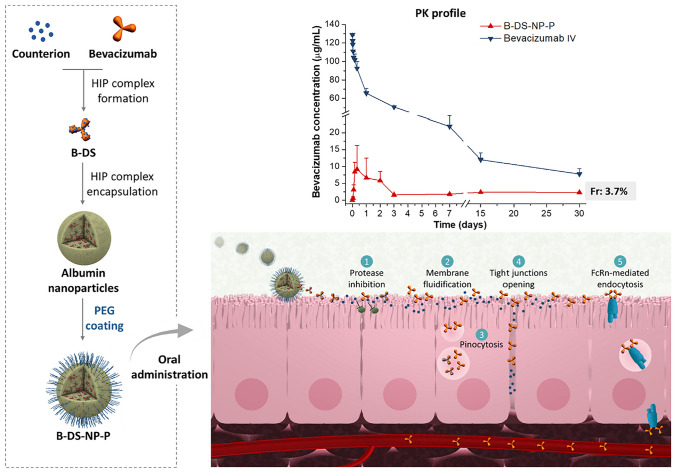

**Supplementary Information:**

The online version contains supplementary material available at 10.1007/s13346-023-01454-0.

## Introduction

In the last decades, therapeutic proteins have played a significant role in the important transformation that the pharmaceutical field has experienced by precision therapeutics, offering targeted and highly specific treatments. These macromolecules, such as monoclonal antibodies and recombinant proteins, have shown great promise in the treatment of various diseases, including cancer, infections, autoimmune disorders, and genetic disorders [1]. Apart from a high efficacy, they often offer a favorable safety profile compared to traditional small molecule drugs [2]. Additionally, therapeutic proteins can act on specific molecular targets, modulate biological pathways, and enhance the body’s natural defense mechanisms, providing novel therapeutic options for previously untreatable conditions [3].

In spite of important advantages, the large majority of these therapies are primarily administered through the parenteral route, which includes intravenous or subcutaneous injections. In general, these administration methods are challenged to be replicated in a non-medical environment [4] and, thus, may limit their accessibility and practicality for patients outside of hospital settings [5]. In the last years, important research efforts are focused on the development of alternative and patient-friendly administration routes for protein delivery (i.e., oral administration).

Oral administration is a non-invasive route and allows patients to self-administer medications easily, improving patient compliance and adherence to the prescribed treatment regimen [6]. This is particularly important for chronic or long-term treatments [7]. Moreover, oral medications are generally more cost-effective compared to other routes of administration, requiring fewer resources for administration, monitoring, and disposal. Despite all of these advantages, oral delivery may not be suitable for all active substances and remains a challenge for therapeutic proteins. These compounds usually show a very poor oral bioavailability that is consequence from their physicochemical properties and the physiological conditions within the gut [8]. In fact, therapeutic proteins are susceptible to inactivation in the harsh conditions of the gastrointestinal tract, characterized by an important variability in the pH conditions and the presence of digestive enzymes along the gut and in the brush border of enterocytes [9]. Moreover, the mucus layer lining the epithelium of the gastrointestinal tract can trap these macromolecules, preventing their arrival to the surface of absorptive cells [10]. Finally, proteins are typically hydrophilic, with a charged nature and large size with (sometimes) complex three-dimensional structures that hinder their efficient absorption through the gastrointestinal tract [11].

In order to solve these drawbacks and to improve the oral absorption and bioavailability of therapeutic proteins, different chemical and pharmaceutical approaches have been proposed. Chemical modifications would involve the alteration of the macromolecule’s structure by introducing a second molecule or a functional group in order to improve its resistance to enzymatic degradation and enhance its absorption across the gastrointestinal tract. Some common chemical modifications of proteins and peptides include the binding of polyethylene glycol chains [12], carbohydrate chains (i.e., glycans [13], fatty acids [14], or methylation [15]). Pharmaceutical strategies may involve the use of formulation approaches, protease inhibitors or permeation enhancers. Protease inhibitors can prevent or reduce the breakdown of therapeutic proteins by luminal secreted enzymes [16] and/or membrane-bound proteases [17], thus improving their absorption and overall efficacy. Absorption enhancers can improve the absorption of proteins by temporarily opening tight junctions between intestinal cells [18].

Within this context, the use of pharmaceutical carriers (i.e., microparticles and nanoparticles) appears to be the most suitable formulation strategy to improve the bioavailability of therapeutic proteins. Nanocarriers offer versatility to both protect the loaded protein against its premature inactivation and capability co-encapsulate protease inhibitors [19] or absorption enhancers [20]. In any case, nanoparticles with the ability to diffuse through the mucus layer would be the most effective device to promote oral delivery of macromolecules. These mucus-permeating properties can be generated by modifying the nanoparticle surface with hydrophilic substances to reduce the interaction with mucins (and other components of the mucosa) and, thus, increase their ability to reach the gut epithelium [21, 22]. The coating of nanoparticles with poly(ethylene glycol) or certain polysaccharides has been effective for this purpose [23, 24].

The aim of this work was to evaluate the ability of protein-based nanoparticles with mucus-permeating properties in combination with permeation enhancers as a dosage form to promote the oral bioavailability of bevacizumab, as a model of therapeutic protein. Bevacizumab is a fully humanized IgG1 monoclonal antibody that specifically binds to and blocks the activity of the vascular endothelial growth factor (VEGF), inhibiting the formation of new blood vessels [25]. For this purpose, hydrophobic ion-pairing complexes between bevacizumab and anionic permeation enhancers (either sodium deoxycholate or sodium docusate) were encapsulated in PEG-coated albumin nanoparticles. These nanoparticles have been shown a good capability to load proteins without compromising their stability [26].

## Materials and methods

### Materials

Human serum albumin, sodium deoxycholate (DS), docusate sodium salt (DOCU), poly(ethylene glycol) 35,000 Da (PEG35), trehalose dihydrate, sodium azide, and agarose were purchased from Sigma-Aldrich (Steinheim, Germany). Bevacizumab (Avastin®) was purchased from Roche (Madrid, Spain). Ethanol absolute was obtained from Scharlab (Sentmenat, Spain). Lumogen® F-Red 305 was supplied by BASF (Ludwigshafen am Rhein, Germany). European bacteriological agar, peptone, LB broth, and agarose were provided by Condalab (Torrejón de Ardoz, Spain). O.C.T.™ Compound Tissue-Tek was obtained from Sakura Finetek Europe (Alphen aan Der Rijn, The Netherlands). Isoflurane was purchased from Braun (Barcelona, Spain). Shikari® Q-BEVA Enzyme Immunoassay used for the detection of bevacizumab was purchased from Matriks Biotek (Gölbaşı, Turkey).

### Hydrophobic ion-pairing (HIP) complex formation with bevacizumab

The hydrophobic ion-pairing (HIP) complexes were prepared by mixing an aqueous solution of bevacizumab with either sodium deoxycholate (DS) or sodium docusate (DOCU) at different bevacizumab-to-counterion ratios. The pH was adjusted to 5, 6.2, or 9. The mixtures were agitated for 15 min at 400 rpm and then centrifuged at 21,000 × *g* for 15 min at 4 °C (Sigma 3K30 Osterode am Harz, Germany). The supernatants were withdrawn, and the amount of bevacizumab was quantified by HPLC. The resulting complex was finally dispersed in water and freeze-dried in a Telstar Lyobeta Mini apparatus (Telstar, Terrassa, Spain).

### Characterization of HIP complex with bevacizumab 

#### Complex formation efficiency

To determine the complex formation efficiency between the monoclonal antibody and the counterion, the amount of free bevacizumab (recovered from the supernatants during the purification step) was quantified by a validated size exclusion chromatography method in an Agilent model 1200 series (Agilent Technologies, Waldbronn, Germany), coupled with a photodiode array detection system at 280 nm. As stationary phase, a Biozen column (3 μm dSEC-2 200 A, 300 × 4.6 mm; Phenomenex, CA, USA) was employed, and the mobile phase was buffer phosphate (35 mM pH 6.8; 150 mM NaCl) in an isocratic mode. The flow rate was 0.2 mL/min, and the temperature of the column was set to 30 °C. The calibration curves were performed using standard solutions of bevacizumab in the range from 22.5 to 300 μg/mL (*R*^2^ > 0.999). The quantification limit for bevacizumab was found to be 12.5 μg/mL. 

The complex formation efficiency (CE, in percentage) was calculated using Eq. (1):

1 $$CE(\%)=\frac{Mi-Mf}{Mi}\times100$$

being Mi the initial amount of bevacizumab and Mf the unbound bevacizumab quantified by HPLC in supernatants obtained after the centrifugation of the complexes.

#### FTIR analysis of bevacizumab-counterion

Fourier transform infrared spectroscopy (FTIR) using a Fourier transform spectrophotometer IR Affinity-1S (Shimadzu, Japan) coupled to a Specac Golden Gate ATR was conducted to analyze the formation of the HIP complex. For the analysis, samples were deposited on the diamond window. Spectra were collected in the mode reflectance under the following conditions: wavenumber from 600 to 4000 cm^−1^ at 2 cm^−1^ of resolution and 50 scans per spectrum. Spectra were analyzed employing the LabSolutions IR software.

#### HIP complex dissociation

The dissociation of HIP complexes was evaluated by incubating samples with 4 mg bevacizumab, under magnetic stirring at 300 rpm, in 5 mL of an aqueous medium (water, gastric simulated fluid at pH 1.6, or intestinal simulated fluid at pH 6.8). After 30 min of incubation, samples were taken and centrifuged at 21,000 × *g* for 15 min at 4 °C (Sigma 3K30 Osterode am Harz, Germany). The amount of bevacizumab in the supernatants was quantified by HPLC, and the percentage of dissociated bevacizumab was calculated as the quotient between amount of bevacizumab in the supernatant and the initial amount of the monoclonal antibody used to form the complex.

### Preparation of empty nanoparticles

Human serum albumin (HSA) nanoparticles, containing either sodium deoxycholate (DS) or sodium docusate (DOCU), were prepared by a desolvation process previously described [26] with minor modifications.

For this purpose, 177 µL of an aqueous solution of DS (25 mg/mL) or 600 µL of DOCU (6 mg/mL) was added to 8 mL of an albumin aqueous solution (12.5 mg/mL) and the pH was adjusted to 5.6 with HCl 1N. Nanoparticles were formed by the addition of 16 mL ethanol under magnetic stirring. Then, 500 µL of an aqueous solution of PEG35 (100 mg/mL) was added dropwise to coat the freshly formed nanoparticles. After the incubation of the mixture for 30 min, the organic solvent was eliminated under reduced pressure (Büchi Rotavapor R-144; Postfach, Switzerland) and the resulting nanosuspensions were purified by centrifugation at 41,000 × *g* for 20 min at 4 °C (Sigma 3K30 Osterode am Harz, Germany). Finally, the pellet containing the nanoparticles was dispersed in an aqueous solution of trehalose (3% w/v) and freeze-dried (Telstar Lyobeta Mini). These nanoparticles were named as DS-NP-P and DOCU-NP-P.

Control nanoparticles without DS or DOCU were also formulated using the same protocol and were identified as NP-P.

### Preparation of Lumogen®-labeled nanoparticles

Nanoparticles were fluorescently labeled with Lumogen® F-Red 305. Briefly, Lumogen® Red was encapsulated into human serum albumin nanoparticles by adding 2.6 mL of a stock solution of the fluorescent tag in ethanol (0.1 mg/mL) to the aqueous solution of HSA before the formation of the nanoparticles. Then, nanoparticles were coated, purified, and freeze-dried as previously described.

### Preparation of bevacizumab-loaded nanoparticles

Bevacizumab was encapsulated in human serum albumin nanoparticles, as HIP complex with either DS (B-DS) or DOCU (B-DOCU). These nanoparticles were obtained as described above. In brief, a variable amount of either B-DS or B-DOCU (corresponding to 8 mg bevacizumab) was added to an aqueous solution of HSA (100 mg in 6 mL purified water). After incubation, the pH was adjusted to 6–6.4 with HCl 1N and the formation of nanoparticles was induced by the addition of 16 mL ethanol. Then, nanoparticles were coated with PEG35 (500 µL of a solution in water 100 mg/mL) before elimination of the organic solvents and purification by centrifugation at 41,000 × *g* for 20 min at 4 °C (Sigma 3K30 Osterode am Harz, Germany). Finally, nanoparticles were freeze-dried using trehalose as cryoprotectant. These formulations were identified as B-DS-NP-P and B-DOCU-NP-P.

Control nanoparticles containing free bevacizumab (B-NP-P) were prepared as described above but in the absence of DS or DOCU. For this purpose, 8 mg bevacizumab was dissolved in the aqueous solution of albumin prior to pH adjustment and desolvation of the protein with ethanol. Uncoated nanoparticles, identified as B-NP, were also prepared in the absence of PEG35.

### Physicochemical characterization of nanoparticles

#### Mean size, polydispersity index (PDI), and zeta potential

Nanoparticles were dispersed in ultrapure water, and the mean particle size and polydispersity index (PDI) were measured by dynamic light scattering (DLS) at a scattering angle of 90° at 25 °C. Electrophoretic light scattering (ELS) was used to determine the zeta potential. For this purpose, nanoparticles were dispersed in ultrapure water. This characterization was carried out in a ZetaPlus analyzer system (Brookhaven Instruments Corporation, Holtsville, NY).

#### Evaluation of shape and morphology

The morphology and shape of nanoparticles were evaluated by SEM. Two milligrams of lyophilized nanoparticles was dispersed in deionized water. The cryoprotectant was eliminated by centrifugation at 1850 × *g* for 20 min at 4 °C. Then, the pellet was redispersed in 2 mL water and 25 µL was deposited on SEM grids. After the drops were dried at room temperature, samples were coated with a gold layer using Emitech K550 Gold Sputter Coater (Quorum Technologies, Laughton, UK). Finally, samples were analyzed using a ZEISS Sigma 500 VP FE-SEM apparatus (Zeiss Microscopy, Jena, Germany).

#### Total process yield

The amount of HSA transformed into nanoparticles was calculated by HPLC as described before. The analytical conditions were as follows: Agilent model 1200 series, photodiode array detection system at 280 nm, Biozen column (3 μm dSEC-2 200 A, 300 × 4.6 mm), and buffer phosphate 35 mM pH 6.8 and 150 mM NaCl. The flow rate and the temperature were the same as described by bevacizumab (0.2 mL/min and 30 °C, respectively). The calibration curves were performed at concentrations ranging from 22.5 to 300 μg/mL (*R*^2^ > 0.9994). Under these conditions, the quantification limit for HSA was found to be 10 μg/mL.

For the quantification, fresh nanoparticles were centrifuged at 41,000 × *g* for 20 min at 4 °C. Then, the supernatants were collected for albumin quantification and the pellets digested in NaOH 0.025 N for total disruption of nanoparticles. The resulting samples were diluted with water for injection and analyzed by HPLC.

#### FTIR and DSC analysis

FTIR analysis of the nanoparticles was performed following the same methodology explained above. The thermal profile of nanoparticles was evaluated in a Differential Scanning Calorimeter TA DSC 25 Discovery series apparatus (TA Instruments, New Castle, DE). For this purpose, between 5 and 10 mg of each sample was weighed in a 40 µL aluminum pan and closed with a hermetic lid assuring good contact between the sample and the capsule bottom. The thermograms were analyzed under an inert nitrogen atmosphere (gas flow, 50 mL/min) from − 40 to 250 °C, employing a ramp heating/cooling rate of 10 °C/min. TRIOS software (TA Instruments, New Castle, DE) was used to analyze the data.

#### Bevacizumab quantification and integrity by microfluidic electrophoresis

Bevacizumab was identified and quantified in an Experion™ Automated Electrophoresis System (Bio Rad, Hercules, CA). For this purpose, 4 mg of each formulation was dispersed in 1 mL of water and centrifuged at 41,000 × *g*, 10 min at 4 °C. The pellets were then digested in NaOH 0.025 N under agitation for 3 min. The resulting samples were finally analyzed under non-reducing conditions according to the manufacturer’s protocol. The obtained electropherograms and simulated gel with densitometric bands were analyzed using the Experion™ software.

#### Bevacizumab quantification by ELISA

Bevacizumab was also quantified by Enzyme-Linked Immunosorbent Assay (ELISA) using the commercial kit Shikari® Q-BEVA (Matriks Biotek Co., Ankara, Turkey). In this case, 10 mg of each formulation was dispersed in NaOH 0.025 N and maintained under agitation for 3 min at room temperature. Samples were then treated in accordance with the manufacturer’s instructions, and the 96-well plate was read at 450/650 nm using a PowerWave HT microplate spectrophotometer (BioTek Instruments, Inc., Winooski, VT).

### Ex vivo mucus diffusion studies by MPT

The diffusion of nanoparticles in pig intestinal mucus was performed as previously described [27, 28]. Briefly, 4 mg Lumogen® Red-labeled nanoparticles (4 mg/mL) was dispersed in 0.5 g mucus and incubated for 2 h at 37 °C at 60 rpm (Labnet VorTemp 56 EVC, Labnet International, Inc., Edison, NJ). The mucus was obtained following the procedure previously described [27, 28]. The movement of nanoparticles was recorded in a two-dimensional plane at 30 frames/s during 10 s by a high-speed camera (Allied Vision Technologies, Stadtroda, Germany) attached to a wide-field epifluorescence microscope used at 63 × magnification oil immersion lens (Leica DM IRB, Wetzlar, Germany). A minimum of 100 trajectories were captured and later tracked and analyzed using an image processing software (Fiji ImageJ).

The diffusion coefficient of the nanoparticles in water (*D*°) was obtained from the Stokes–Einstein equation [28], whereas the “Effective Diffusion Coefficient” (< Deff >) was calculated as follows:

2 $$Deff=\frac{<MSD>}{4\cdot\Delta t}$$

in which < MSD > is the mean square displacement of 100 individual trajectories, 4 is a constant related to the 2-dimensional mode of video capture, and Δ*t* is the selected time interval. All the formulations were expressed as the ratio (%) between their Deff and their *D*° (diffusions in mucus and in water, respectively).

### Biodistribution of nanoparticles within the gut

The biodistribution in vivo of nanoparticles was evaluated in healthy Wistar rats (Envigo, Indianapolis, IN). The protocol, previously described [24, 28], was approved by the “Ethical and Biosafety Committee for Research on Animals” at the University of Navarra in accordance with the European legislation on animal experimentations (protocol number 045–18).

For the study, fasted animals received by oral gavage 25 mg of fluorescently labeled nanoparticles dispersed in 700 µL water. As control, a suspension of Lumogen Red in water containing Tween 80 (0.2% w/v) was employed. After 4 h of administration, the rats were anesthetized with isoflurane by inhalation and sacrificed. The gastrointestinal tract was collected, and small portions of the stomach, small intestine, and cecum were obtained, cleaned with PBS and embedded in O.C.T.™, and frozen. For analysis, each portion was cut into 5 μm sections on a cryostat and attached to glass slides before staining with DAPI for 15 min [24]. Samples were visualized in an Automated Microscope Zeiss Axio Imager M1 with an Axiocam MRm camera (Zeiss Microscopy, Jena, Germany). The images were processed by Fiji ImageJ.

### In vivo evaluation in *Caenorhabditis elegans*

#### Strain and culture condition

*C. elegans* transgenic strain FT63 labeled with green fluorescent protein (GFP) at the epithelial junctions (DLG::GFP) was obtained from the Caenorhabditis Genetics Center (CGC, University of Minnesota, MN). Worms were cultured at 20 °C on NGM (Nematode Growth Medium) agar with *E. coli* OP50 as normal nematode feed source. For all experiments, age-synchronized worms were employed. For this purpose, worms were treated with sodium hypochlorite and then incubated for at least 24 h in M9 buffer solution until the eggs hatch into larvae. Then, approximately 500 L1 larvae were transferred to plates and cultured to a defined age in each experiment.

#### Analysis of *C. elegans* epithelialjunctions 

The assay was carried out in a NGM supplemented with different treatments including nanoparticles (10 mg/mL) and DS and DOCU (0.13 mg/mL). The concentrations of the free components (DS or DOCU) corresponded to the theoretical amounts presented in the nanoparticles.

At L1, 100 worms were placed in each well and incubated at 20 °C. Once the worms achieved the L4 stage, they were collected and fixed on a 2% agarose and 1% sodium azide glass [29]. Samples were visualized using an Automated Microscope Zeiss Axio Imager M1 with an Axiocam MRm camera (Zeiss Microscopy, Jena, Germany).

The criteria for the intestinal disruption were based on the fluorescence of the epithelial cells. The epithelial cells of worms without disruption were visualized forming a ladder, whereas when the disruption occurred, the fluorescence of the GFP becomes blurry or fragmented.

#### Lifespan assay

The lifespan assay was conducted in L4 larva stage seeded in NGM plates containing 40 mM 5-fluoro-2′-deoxyuridine [30]. In all cases, 25 worms were placed in each well and each treatment was evaluated in triplicates and kept at 20 °C. Dead worms were counted and removed every 2 days until day 15, when counting was daily until the end of the study. Nematodes were considered dead if they did not move after repeated mechanical stimuli.

### In vitro release study

In vitro release studies of bevacizumab from different nanoparticles were carried out in simulated gastric (SGF, pH 1.2) and intestinal fluids (SIF, pH 6.8). For each time point, 20 mg of bevacizumab-loaded formulations was dispersed in 1 mL SFG and placed in a shaking bath (37 °C) with a constant agitation of 60 strokes/min (VorTemp 56, Labnet International, Edison, NJ). At predetermined time intervals, samples were centrifuged for 10 min at 41,000 × g (Rotor 3336, Biofuge Heraeus, Hanau, Germany). After 30 min in SFG, the samples were centrifuged, and the pellets were redispersed in SIF. Supernatants were analyzed by HPLC.

### In vivo pharmacokinetic study in rats

The pharmacokinetic study was performed in healthy male Wistar rats (Envigo, Indianapolis, IN). All the manipulations were carried out following an approved protocol by the “Ethical and Biosafety Committee for Research on Animals” from the University of Navarra, following the European legislation on animal experimentation (protocol 113–21). At the arrival of the rats, they were disposed in their cages with free access to food and water. The conditions of the animal house were strictly controlled with 12-h dark/light cycles under controlled temperature (23 ± 2 °C). Rats were not manipulated until 7-day acclimation process had finished. After this period, rats were randomly divided into groups of 6 animals each. Before the beginning of the experiment, rats were fasted 12 h before the start with free access to water. Different treatment of nanoparticles was then administered by oral gavage to rats at a dose of 15 mg/kg of bevacizumab. The dose of the intravenous administration was 5 mg/kg of bevacizumab. At different time points, blood samples for the tail vein were obtained. Concentrations of bevacizumab in plasma were quantified by the Shikari® Q-BEVA ELISA kit. The bevacizumab concentrations obtained were then represented versus time, and a pharmacokinetic analysis was performed using PKSolver [31]. Maximal plasma concentration (*C*_max_), time in which *C*_max_ is reached (*T*_max_), area under the concentration–time curve from time 0 to last sampling time (AUC), and clearance (Cl) were calculated. Also, the relative bioavailability (Fr %) was calculated as follows:

3 $$\text{Fr}\left({\%}\right)\text{=}\frac{\text{AUC oral}\times\text{ Dose i.v.}}{\text{AUC i.v.}\times\text{Dose oral}}\times100$$

### Statistical analysis

The means and standard errors were calculated for every dataset. All the group comparisons and statistical analyses were performed using a one-way ANOVA test followed by a Tukey–Kramer multicomparison test. In all cases, *p* < 0.05 was considered as a statistically significant difference. All calculations were performed using GraphPad Prism v6 (GraphPad Software, Boston, MA), and the curves were plotted with the Origin 8 software from Origin Lab (Northampton, MA).

## Results

### Characterization of HIP complex with bevacizumab

**Fig. 1:**
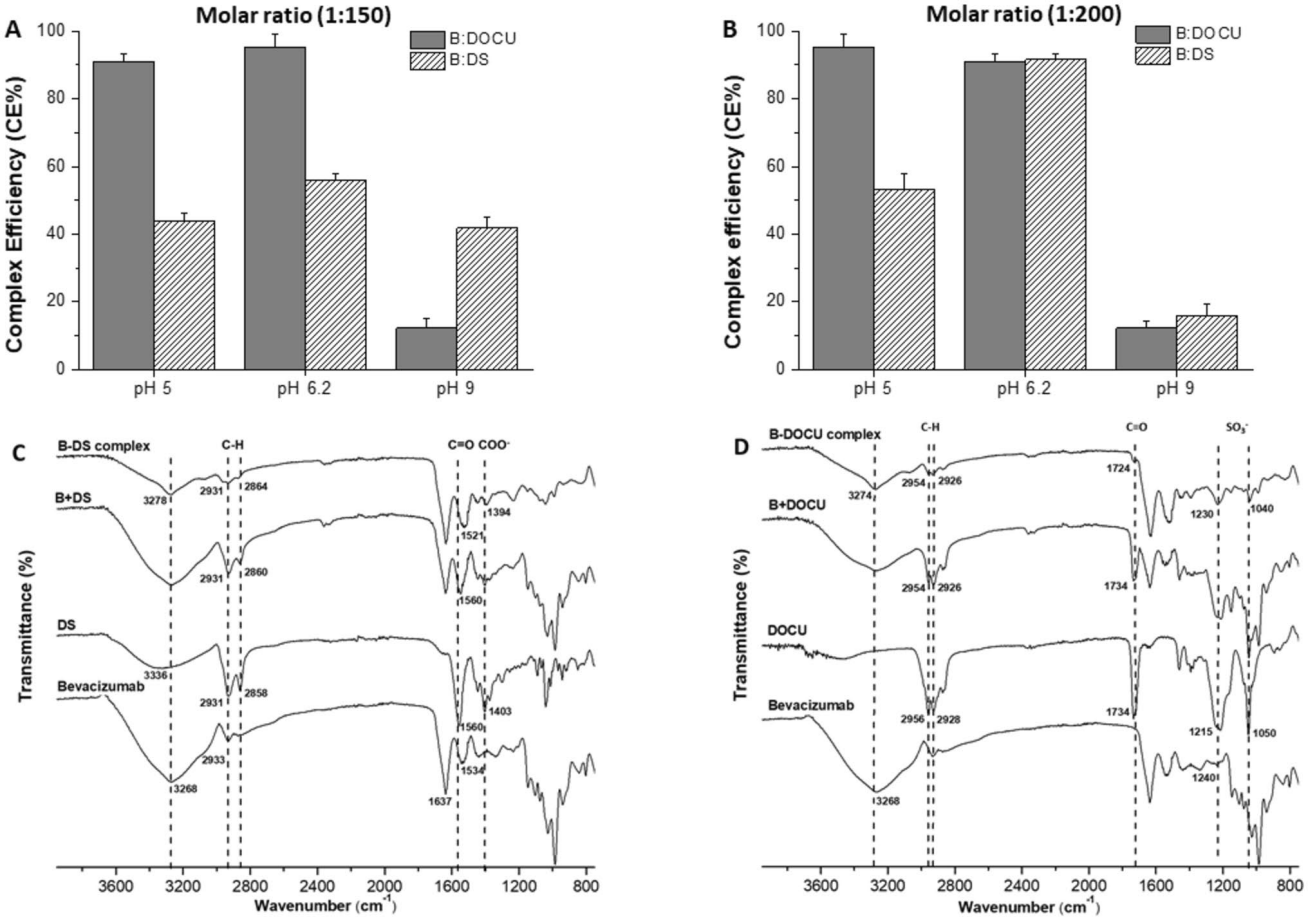
Complex formation efficiency between bevacizumab and either DS or DOCU at different pH conditions and bevacizumab-to-counterion molar ratios of 1:150 (**A**) and 1:200 (**B**). The isoelectric point (pI) of bevacizumab is approximately 8.3 [25]. Data expressed as mean ± SD (*n* = 3). Bevacizumab was quantified by HPLC. FTIR spectra of HIP complex between bevacizumab and DS (**C**) or DOCU (**D**). Dashed lines correspond to the bevacizumab N–H group (3268 cm^*−*1^), the alkane groups CH_2_ and CH_3_ (C-H; 2930 and 2860 cm^*−*1^), the carboxylic group of DS (COO^*−*^; 1403 cm^*−*1^), the carbonyl group of DOCU (C = O, 1733 cm^*−*1^), and the sulfonate group of DOCU (SO_3_^*−*^; 1215 and 1050 cm^*−*^^1^)

In this work, bevacizumab complex with either DS or DOCU was prepared at two different molar ratios (1:150 and 1:200). In these conditions, the complex formation efficiency between bevacizumab and DOCU was close to 90% at pH values of 5 and 6.2 and lower than 20% at pH 9 (Fig. 1A, B). For the bevacizumab-DS complex, the highest CE value (about 90%) was observed at a molar ratio of 1:200 and pH 6.2. Overall, the complex formation efficiency was higher for DOCU-based complex than for DS-based ones.

FTIR was conducted to evidence the interactions between the monoclonal antibody and either DS (Fig. 1C) or DOCU (Fig. 1D). For the B-DS complex, the interaction between the monoclonal antibody and the counterion was confirmed mainly by the shift of the signal corresponding to the carboxylic group of DS from 1403 to 1394 cm^−1^, as well as by the broadening and intensity reduction associated to O–H stretching vibrations and C = O groups of bevacizumab and DS upon complexation. For B-DOCU complex, the signals associated to the sulfonate group of docusate (1215 cm^−1^ and 1050 cm^−1^) and the C-N vibration of bevacizumab (1240 cm^−1^ and 1145 cm^−1^) turned into a pronounced single band at 1230 cm^−1^ and 1040 cm^−1^, respectively, when the complex was formed. All of these modifications provided evidence of interaction between protonated amino acids of bevacizumab and the negative ionized groups of DS (COO^−^) and DOCU (SO_3_^−^).

HIP complexes between the monoclonal antibody and DS or DOCU were also incubated for 30 min in different media and, after the centrifugation, the amount of bevacizumab in the supernatants was quantified (Supplementary information, Fig. S1). In water, the amount of dissociated bevacizumab was lower than 10% for both complexes. On the contrary, a different behavior for B-DS and B-DOCU was observed when analyzed in simulated gastric and intestinal fluids. Thus, for B-DS, about 90% of bevacizumab appeared to be dissociated when incubated in both SGF and SIF for 30 min. On the contrary, less than 10% of bevacizumab was found dissociated for the B-DOCU complex when incubated in either SGF or SIF.

### Characterization and evaluation of albumin nanoparticles

#### Physicochemical characterization

Albumin nanoparticles were prepared by desolvation and then stabilized by their coating with PEG 35,000. These nanoparticles (NP-P) displayed a mean size of about 207 nm with a negative zeta potential of − 25 mV and a yield (calculated as the amount of HSA transformed into nanoparticles) close to 80% (Table 1). The incorporation of either DS or DOCU in PEG-coated nanoparticles significantly increased the mean size of the resulting nanoparticles (DS-NP-P and DOCU-NP-P, respectively). This increase was particularly high for DOCU-NP-P 100 nm bigger than NP-P. DS-NP-P and DOCU-NP-P displayed similar negative zeta potential and yield values as control ones.

**Table 1:** Physicochemical characterization of albumin nanoparticles. Data expressed as mean ± SD (*n* = 6)

	**Size (nm)**	**PDI**	**Ζeta potential (mV)**	**Amount of HSA (%)**
**NP-P**	207 ± 2	0.10 ± 0.01	− 26 ± 1	79 ± 4
**DS-NP-P**	280 ± 13	0.08 ± 0.04	− 28 ± 1	83 ± 3
**DOCU-NP-P**	310 ± 9	0.07 ± 0.01	− 32 ± 4	81 ± 2

**Fig. 2:**
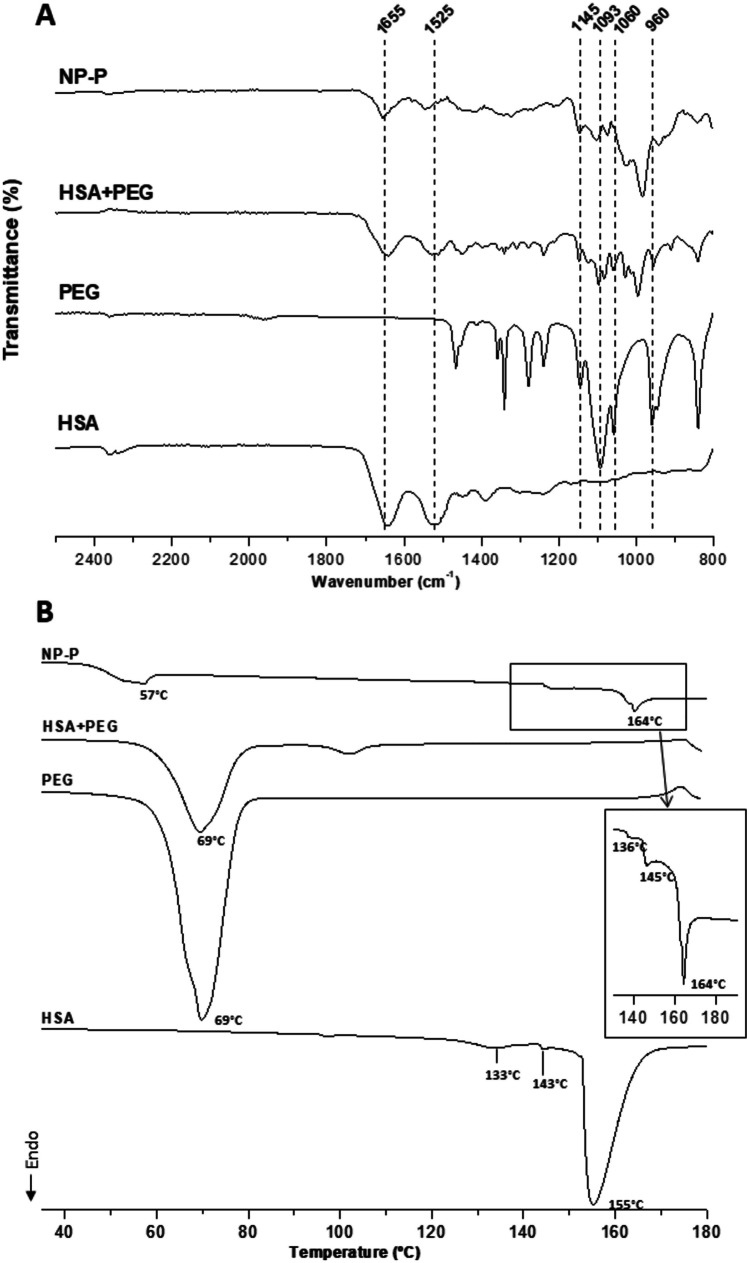
FTIR spectra and DSC curves of PEG-coated nanoparticles (NP-P) and their individual components. In FTIR spectra (**A**), dashed lines correspond to amide I and II of albumin (1655 and 1525 cm^*−*1^, respectively) and the following PEG35 characteristic bands: C-O (1145 cm^*−*1^), C-O stretching band of the OH end group (1093 cm^*−*1^), C–O–C (1059 cm^*−*1^), and CH_2_ (960 cm^*−*^.^1^). In **B**, the upper right box shows a magnification of the spectrum area of NP-P corresponding to the unfolding and melting process of HSA. HSA + PEG: physical mixture of albumin and PEG35

Figure 2A shows the FTIR spectra of PEG-coated albumin nanoparticles (NP-P) and their main individual components. In the nanoparticles, the interaction between PEG35 and albumin was evidenced by the signals observed in the so-called fingerprint region by FTIR analysis (Fig. 2A). Thus, the C-O stretching vibration band of the OH end group and the signal associated to C–O–C stretching vibration of PEG35 were shifted toward higher frequencies (from 1093 cm^−1^ and 1059 cm^−1^, for pure PEG35, to 1103 cm^−1^ and 1074 cm^−1^, in nanoparticles, respectively). In addition, the signals corresponding to CH_2_ groups of PEG35 (960 and 840 cm^−1^) also appeared as slightly broadening bands in the nanoparticles. All of these changes would be the result of the interaction between PEG35 and albumin at the surface of nanoparticles. Figure 2B shows the thermograms obtained by DSC analysis of PEG-coated albumin nanoparticles (NP-P) and their main individual components. For PEG 35,000, the thermogram was characterized by a broad endothermic signal, corresponding with the melting point of this substance (about 68 °C), typical of a no high crystalline substance. This signal was also clearly observed in the physical mixture between albumin and PEG35. Regarding the thermogram of PEG-coated nanoparticles, the broad endothermic peak appearing at 57 °C may be attributed to interaction between PEG35 and albumin. In a similar way, the weak signals observed at 136 °C and 145 °C, as well as a melting endothermic peak detected at higher temperatures (164 °C) compared to HSA (155 °C), would correspond to the protein thermal behavior (unfolding and melting processes) due to its interaction with PEG35.

#### Evaluation of mucus-permeating properties

**Fig. 3:**
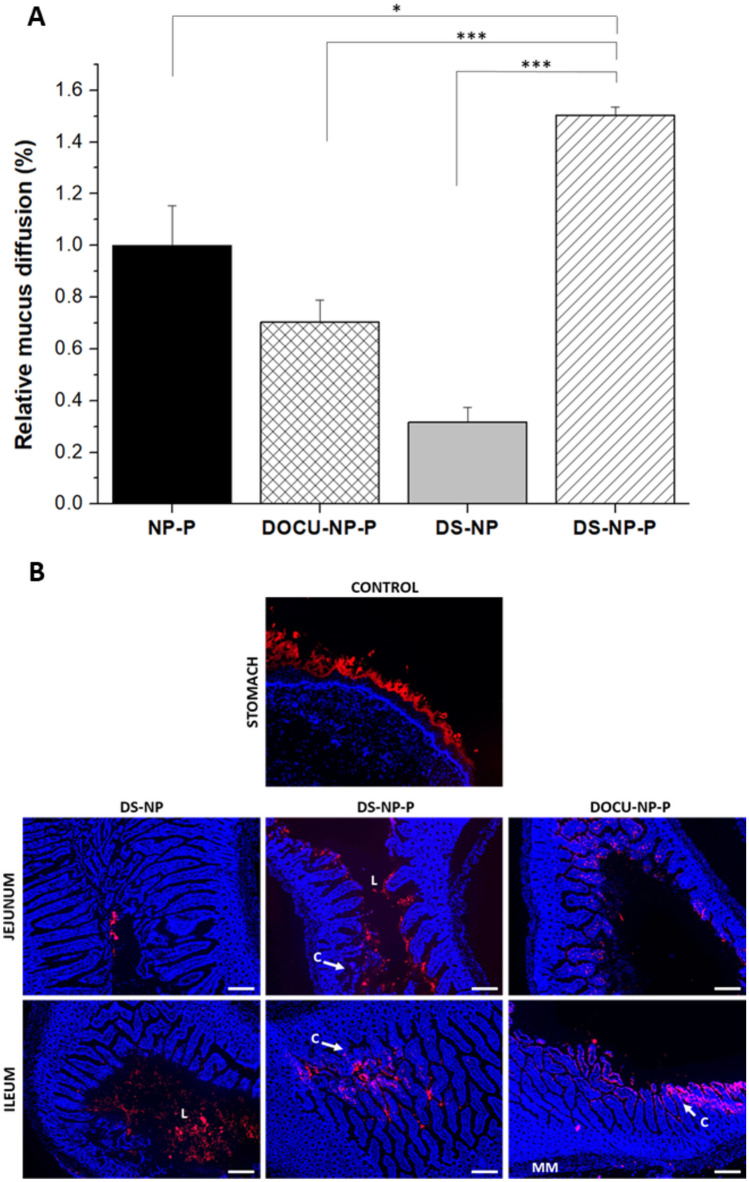
Evaluation of the capability of nanoparticles to diffuse in intestinal mucus and reach the epithelial surface in the gut. **A** The diffusion of nanoparticles was evaluated in pig intestinal mucus. Data are normalized to the value of NP-P and expressed as mean ± SD (*n* = 3); *p < 0.05, *** *p* < 0.001. **B** Fluorescence microscopy images of the gastrointestinal tract after 4 h of administration of Lumogen® Red-labeled nanoparticles containing DS or DOCU (in red). Nuclei of cells are stained with DAPI (blue). L: lumen; MM: muscularis mucosa; C: crypts. Scale bar represents 200 µm

The relative mucus diffusion of the nanoparticles was evaluated in pig intestinal mucus (Supplementary material Table S1 and Fig. 3A). As control, DS-NP (prepared without the outer PEG35 coating layer) was employed. These nanoparticles displayed a mean size of 264 ± 14 nm and a negative zeta potential (− 28 ± 1 mV). PEG-coated nanoparticles containing DS (DS-NP-P) significantly increased the diffusivity in mucus compared with NP-P. Moreover, the presence of PEG35 increase 4.8 times the diffusion for DS-NP-P compared to DS-NP. On the contrary, the incorporation of DOCU to PEG-coated nanoparticles (DOCU-NP-P) decreased 0.7-fold their diffusion in mucus.

Figure 3B shows the fluorescence micrographs of the gastrointestinal tract of rats obtained 4 h post-administration of the different Lumogen® Red-loaded nanoparticles. Likely, the Lumogen suspension (control) was mainly localized in the stomach. Also, as a control, DS-NP (without PEG35) was used. PEG-coated and uncoated nanoparticles depicted differences that may be attributed to the presence of PEG35. In this case, the localization of DS-NP seemed to be almost limited to the lumen and the mucus layer. On the contrary, PEG-coated nanoparticles (DOCU-NP-P and DS-NP-P) were able to reach the intestinal epithelium occupying the intervilli spaces and even reaching the intestinal crypts especially at distal portion of the small intestine (jejunum and ileum).

#### Evaluation of the permeation enhancer properties

**Fig. 4:**
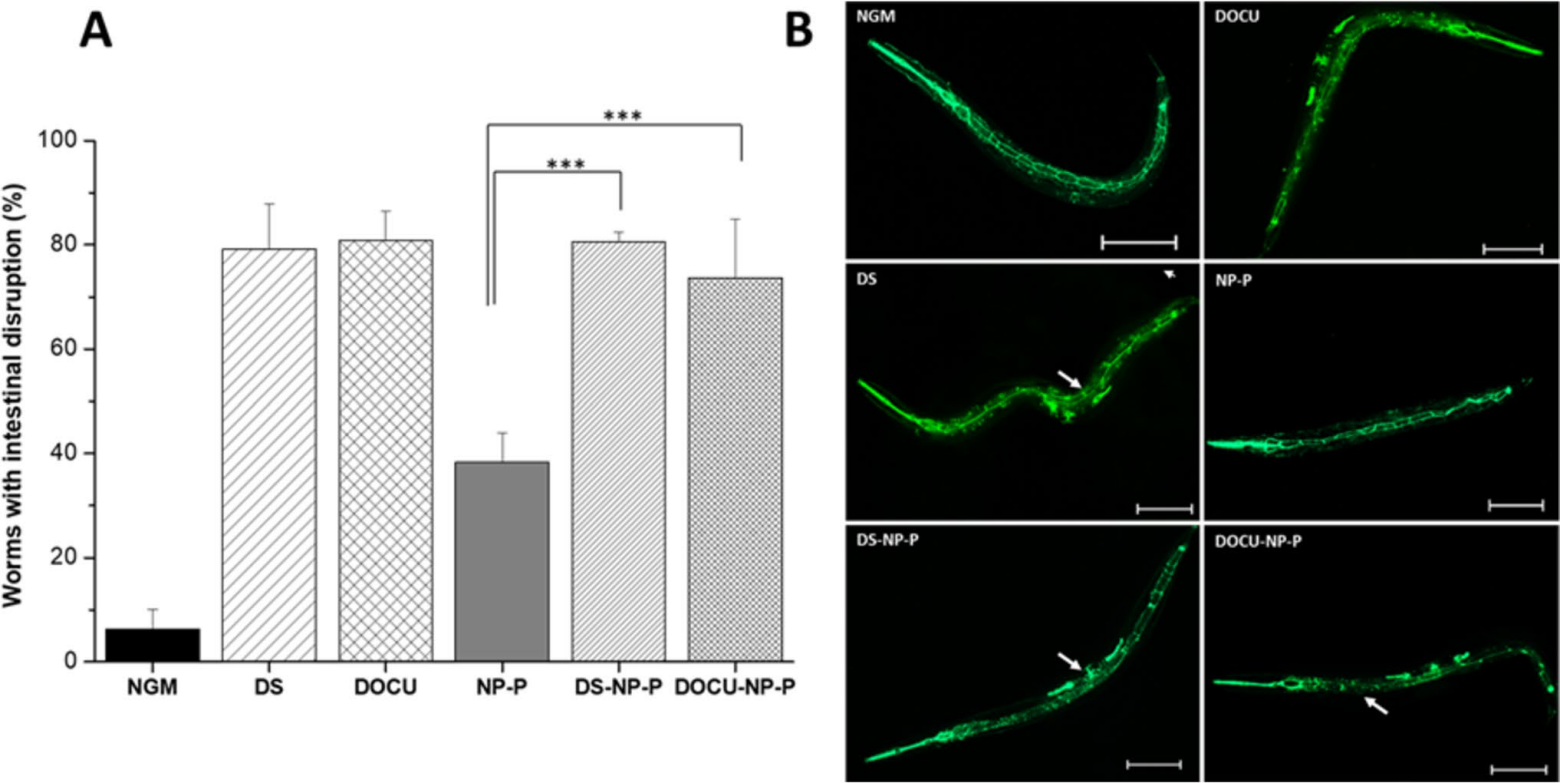
Evaluation of the permeation enhancer effect of nanoparticles and the lifespan of transgenic *C. elegans* FT63 (DLG:GFP). **A** Effect on the disruption capacity of nanoparticles at the epithelial junctions of the intestine of transgenic *C. elegans*. Data expressed as mean ± SD (*n* > 90). *** *p* < 0.001. **B** Images of disruption on intestinal epithelium of worms taken in a fluorescence microscope 10 × at L4 stage. Scale bar represents 100 µm. DS: free sodium deoxycholate, DOCU: free sodium docusate, NP-P: PEG-coated albumin nanoparticles, DS-NP-P: DS encapsulated into PEG-coated albumin nanoparticles, DOCU-NP-P: DOCU encapsulated into PEG-coated albumin nanoparticles. NGM: control

After confirming that the worms ingested nanoparticles orally (Fig. S2 in Supplementary information), the effect of nanoparticles and the permeation enhancers (DS and DOCU) on the integrity of the epithelial junctions and lifespan was evaluated in FT63 worms. As expected, the supplementation of NGM with either DS or DOCU induced a clear loss of the integrity in the intestinal epithelium of animals (Fig. 4A, B). However, the incidence in the number of worms that showed intestinal disruption was similar for animals treated with free or nanoencapsulated absorption enhancers. In the same way, no statistically significant differences were observed between the treatments with DS or with DOCU (Fig. 4A). In contrast, NP-P (without permeation enhancer) displayed an approx. 2 times lower incidence of animals with intestinal disruption than DS-NP-P or DOCU-NP-P (*p* < 0.001).

Results from the lifespan experiment are summarized in Table 2. The Kaplan–Meier representations of the percentage of worms alive over time can be found in the Supplementary information (Fig. S3). In all cases, the lifespan of worms showed similar values to control (NGM group), independent of the treatment. The overall median survival was 17 days and the maximum life expectancy close to 25 days.

**Table 2:** Effect of permeation enhancers, free or nanoencapsulated, supplementation on the lifespan of *C. elegans*, expressed as mean, median, and maximum life expectancy. NGM: control, *n* ≥ 70 worms

**Treatment**	**Mean survival (days)**	**Median survival (days)**	**Maximum (days)**
**NGM**	13	16 ± 1	24 ± 2
**DS**	13	18 ± 3	23 ± 1
**DOCU**	17	20 ± 1	25 ± 2
**DS-NP-P**	15	16 ± 2	26 ± 2
**DOCU-NP-P**	13	15 ± 1	24 ± 2

### Bevacizumab-loaded nanoparticles

#### Characterization of nanoparticles

Table 3 summarizes the main physicochemical characteristics of bevacizumab-loaded nanoparticles employed in this study. When free bevacizumab was incorporated in albumin nanoparticles, the mean size was about 225 nm and the zeta potential close to − 36 mV. The coating of those nanoparticles with PEG35 (B-NP-P) increased the mean size (235 nm) and slightly reduced the negative zeta potential (about − 28 mV). On the other hand, the encapsulation of HIP complexes in albumin nanoparticles increased the mean size, until 270 nm (for B-DS-NP-P when B-DS was employed) and 328 nm (for B-DOCU-NP-P, for B-DOCU). In detail, the best encapsulation efficiency (EE) was obtained using the B-DS complex with about 90% of the monoclonal antibody encapsulated and a payload of 43 µg bevacizumab per mg nanoparticle; this EE was significantly higher (*p* < 0.05) than for B-DOCU with a bevacizumab loading of 35 µg/mg, which corresponded to an EE of 70%.

**Table 3:** Physicochemical characterization of bevacizumab-loaded albumin nanoparticles. *PDI* polydispersity index, *EE* encapsulation efficiency, *B-NP* bevacizumab loaded in albumin nanoparticles, *B-NP-P* bevacizumab-loaded nanoparticles coated with PEG35, *B-DS-NP-P* albumin nanoparticles containing the bevacizumab-DS complex coated with PEG35, *B-DOCU-NP-P* albumin nanoparticles containing the bevacizumab-DOCU complex coated with PEG35. Data expressed as mean ± SD (*n* = 6). Bevacizumab was quantified by ELISA

	**Size (nm)**	**PDI**	**Zeta potential (mV)**	**EE (%)**	**Payload (µg/mg NP)**
**B-NP**	225 ± 5	0.06 ± 0.02	− 36 ± 2	82 ± 2	61 ± 3
**B-NP-P**	235 ± 2	0.12 ± 0.03	− 28 ± 4	89 ± 5	46 ± 4
**B-DS-NP-P**	270 ± 6	0.13 ± 0.03	− 29 ± 4	90 ± 2	43 ± 1
**B-DOCU-NP-P**	328 ± 32	0.19 ± 0.08	− 26 ± 5	70 ± 6	35 ± 3

**Fig. 5:**
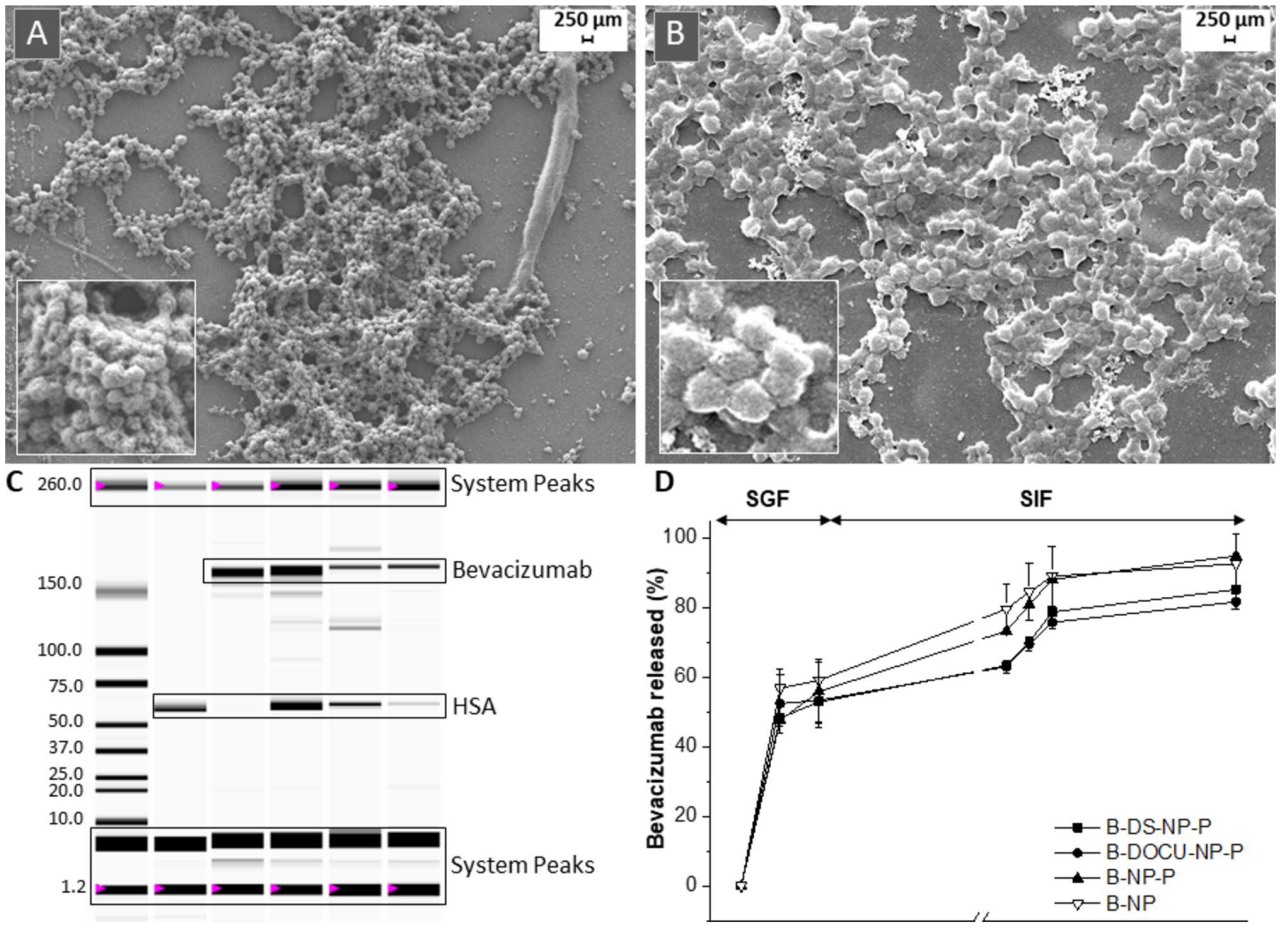
Scanning electron microscope images of B-NP-P (**A**) and B-DS-NP-P (**B**). **C** Microfluidic-based automated electrophoresis of nanoparticles. L: ladder; 1: HSA; 2: bevacizumab; 3: physical mixture (HSA + BEVA); 4: B-DS-NP-P; 5: B-DOCU-NP-P. **D** In vitro release profile of bevacizumab-loaded nanoparticles. Data are expressed as mean ± SD (*n* = 3). Bevacizumab was quantified by HPLC

The morphological analysis by scanning electron microscopy (SEM) showed that all the formulations consisted of a homogeneous population of spherical-shaped nanoparticles (Fig. 5A, B). The size values obtained by this technique were similar to those obtained by dynamic light scattering. Figure 5C shows the microfluidic electrophoresis analysis of these nanoparticles. By this technique, bevacizumab appeared as a band at 165 kDa. The quantification of this band provided bevacizumab loading and EE values similar to that obtained by ELISA (Table 3) confirming that the preparative process of nanoparticles did not have any significant influence on the stability and structural integrity of bevacizumab. Figure 5D shows the release of bevacizumab from nanoparticles during their incubation in SGF (for the first 30 min) and then in SIF. All the formulations displayed a similar profile, characterized by an important release of the bevacizumab content (of about 50–60%) when incubated in SGF. However, when the formulations were moved to the SIF, nanoparticles containing the bevacizumab complexes with either DOCU or DS displayed a lower amount of bevacizumab released than from B-NP or B-NP-P. Thus, after 8 h of incubation in SGF (for 30 min) and SIF (for 7.5 h), the amount of bevacizumab released from B-NP or B-NP-P was about 90% of the initial content, whereas for B-DOCU-NP-P or B-DS-NP-P, the amount of the monoclonal antibody released was only about 75%.

#### In vivo pharmacokinetic study

For the pharmacokinetic study, a bevacizumab solution intravenously administered at a dose of 5 mg/kg was used as control. The plasma levels were characterized by an initial rapid decrease during the first 24 h, followed by a slow decrease during the following 29 days. The pharmacokinetic parameters, summarized in Table 4, showed a *C*_max_ of about 129 µg/mL and an AUC of 659 μg/mL per day.

**Table 4:** Pharmacokinetic parameters obtained for the administration of free and nanoencapsulated bevacizumab at 15 mg/kg. Data are expressed as mean ± SD (*n* = 6). *Bevacizumab* bevacizumab aqueous solution, *B-NP-P* bevacizumab-loaded nanoparticles coated with PEG35, *B-DS-NP-P* albumin nanoparticles containing the bevacizumab-DS complex coated with PEG35, *po* per oral, *T*_*max*_ time to reach maximum plasma concentration, *C*_*max*_ maximum plasma concentration, *t*_*1/2*_ half-life, *AUC* area under the curve, *Fr* relative oral bioavailability, *NA* not available

	***t***_**max**_ **(days)**	***C***_**max**_ **(μg/mL)**	***t***_**1/2**_ **(days)**	**AUC**_**0-30d**_ **(μg/mL·d)**	**Fr**
**Bevacizumab iv**	0	129 ± 1	13 ± 3	659 ± 31	100
**Bevacizumab po**	NA	NA	NA	NA	NA
**B-NP-P**	0.33 ± 0.05	0.08 ± 0.02	NA	0.06 ± 0.02	0.003
**B-DS-NP-P**	0.25 ± 0.08	10 ± 1	36 ± 14	73 ± 7	3.7

**Fig. 6:**
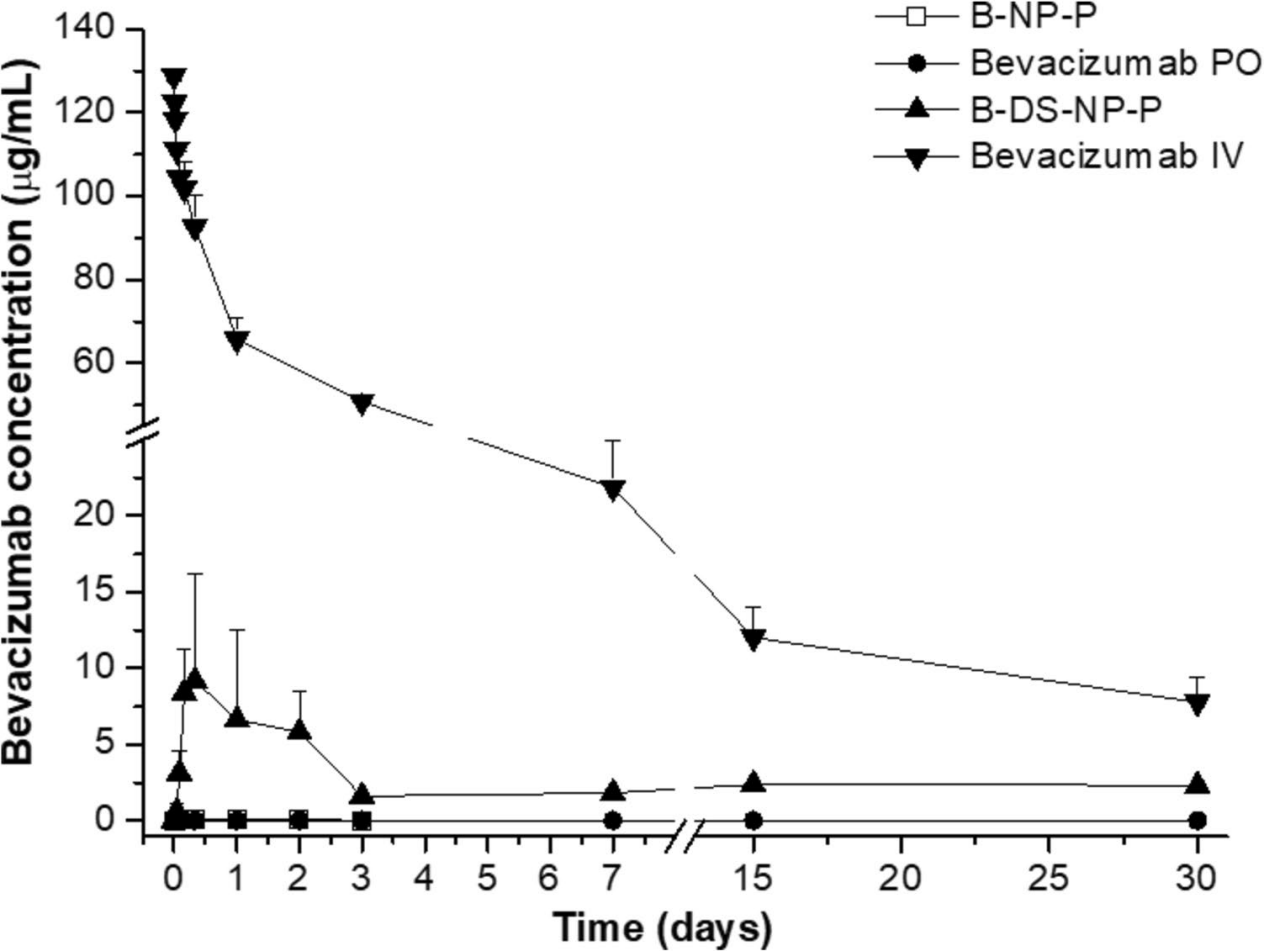
Pharmacokinetic profile of intravenously administered bevacizumab (bevacizumab IV) after a single dose of 5 mg/kg and bevacizumab concentrations after a single oral administration of different formulations at a dose of 15 mg/kg. Data are expressed as mean ± SD (*n* = 6). Bevacizumab: bevacizumab aqueous solution; B-NP-P: bevacizumab-loaded nanoparticles coated with PEG35; B-DS-NP-P: albumin nanoparticles containing the bevacizumab-DS complex coated with PEG35. Bevacizumab was quantified by ELISA

On the other hand, B-NP-P and B-DS-NP-P were orally administered as a single dose of 15 mg/kg (Fig. 6). The plasma levels of bevacizumab after its administration as an oral solution or encapsulated in B-NP-P were very low (less than 70 ng/mL). On the contrary, for B-DS-NP-P, the pharmacokinetic profile of bevacizumab was characterized by an initial rapid increase of its concentration in plasma levels, followed by a slow decrease during the following 3 h, and a plateau of bevacizumab plasma concentration (close to 2 µg/mL) up to 30 days post-administration. The maximum concentration was about 10 μg/mL and the AUC was calculated to be 37 μg/mL per day, with a relative oral bioavailability of 3.7%.

## Discussion

The purpose of the work was to design and evaluate an adequate oral delivery system to promote the oral bioavailability of therapeutic proteins, using bevacizumab as a model. The strategy was based on the encapsulation of the monoclonal antibody as HIP complex with a counterion (either DS or DOCU), in mucus-permeating nanoparticles. Both compounds, DS and DOCU, have been employed to increase the hydrophobicity of proteins (and other drugs) to enhance their encapsulation in SEDDS [32] and nanoparticles based on hydrophobic polymers such as PLGA [33] or lipids [34]. Moreover, DS and DOCU also possess permeation enhancer properties by disrupting in a reversible way the tight junctions between epithelial cells [8, 35], leading to an increase in paracellular transport of drugs through the intercellular gaps [36].

In our case, the complex formation efficiency was higher for the bevacizumab-DOCU complex than for bevacizumab-DS one (Fig. 1). In addition, the formation of the complex was affected by the pH of the medium (particularly for DS-based complex); however, in both cases, the efficiency of the formation process was higher than 90% (molar ratio of 1:200 and pH 6.2). These experimental conditions are in line with previous results in which hydrophobic ion-pairing complexes of bevacizumab with sodium docusate have been prepared (bevacizumab-to-counterion ratio of 1:150 in water) [37, 38]. The dissociation of the two HIP complexes (B-DS and B-DOCU) was evaluated in different media (Supplementary information, Fig. S1). Negligible dissociation was observed for both complexes in water. However, in simulated stomach and intestinal fluids, bevacizumab-DOCU complex showed a lower dissociation than bevacizumab-DS complex. This fact would be related to the strongest interaction of cationic charges (bevacizumab) with sulfate groups (sodium docusate) than with carboxylic groups (sodium deoxycholate) [39]. Similar incomplete dissociation in simulated fluids has been reported for complexes between sodium dodecyl sulfate and a IgG-Fab fragment [40] or octreotide [41].

Next, these complexes were encapsulated in albumin nanoparticles. The mean size was higher for B-DOCU-NP-P than for B-DS-NP-P, whereas the bevacizumab loading was about 20% lower for nanoparticles containing B-DOCU than B-DS complexes (Table 3). The nanoparticles were coated with PEG35 to both confer structural stability and provide a hydrophilic surface without ionizable groups. The intermolecular interactions between albumin and PEG35 on the surface of nanoparticles were evidenced by FTIR and DSC techniques (Fig. 2). Furthermore, the coating of nanoparticles with PEG35 increased the capability of nanoparticles to diffuse in pig intestinal mucus (Fig. 3A), particularly DS-NP-P, which showed a significantly higher diffusivity in mucus (× 5) than their uncovered counterparts (DS-NP). The mucus-permeating properties of PEG-coated nanoparticles (DS-NP-P or DOCU-NP-P) were also evidenced in vivo (Fig. 3B), being capable of reaching the intestinal epithelium surface, whereas uncoated nanoparticles (DS-NP) were found mainly in the mucus layer of the jejunum and ileum (Fig. 3B). These results agree well with previous works employing PLGA [42] or zein nanoparticles [28]. The in vitro release study was performed in simulated gastrointestinal fluid and established a role for hydrophobic complexation, as bevacizumab release was slower when encapsulated as HIP complex with either DOCU or DS. Similar behavior has been previously reported by Peira et al. [38], who demonstrated that the amount of bevacizumab released from lipid nanoparticles decreased when formulated as HIP complex with sodium docusate. PEG coating had no effect in the release, although its role could be more important in the presence of enzymes as its protection of proteins against enzymatic degradation has been previously described [43].

Despite the interest of permeation enhancers to improve the oral absorption of proteins, their safety may be an important concern, particularly when high doses are employed or in chronic treatments. Thus, the disruption of the tight junctions may induce intestinal barrier dysfunction impairing physiological functions [44] and facilitating the passage of unwanted substances (i.e., toxins or pathogens) [45]. Therefore, the ability of nanoparticles containing DS or DOCU to induce the opening of tight junctions and their effect on lifespan was evaluated on *C. elegans* FT63 strain (Fig. 4). This model of intestinal permeability and barrier disruption evaluation has been previously performed for a mechanistic understanding of the effects caused by microorganisms and chemicals [46]. These two compounds clearly altered the integrity of the gut of animals and their effect (measured as the percentage of worms that showed intestinal disruption) was not lost by their encapsulation in albumin nanoparticles (Fig. 4A, B). In any case, and in spite of the disruptive effect observed for DS and DOCU (free or nanoencapsulated), the lifespan of worms was similar to that of the control group (Table 2 and Fig. S3 in Supplementary information) and the reported value in the literature, around 2–3 weeks (14–21 days) [47].

In the pharmacokinetic study in rats (Fig. 6 and Table 4), the administration of bevacizumab encapsulated in PEG-coated nanoparticles (B-NP-P) produced a relative oral bioavailability value of 0.003%. However, when the monoclonal antibody was encapsulated in albumin nanoparticles in the form of HIP complex with DS (B-DS-NP-P), the relative oral bioavailability increased (at least) 1000-fold (3.7%, Table 4).

It is well established that only very small amounts of intact immunoglobulins may reach the systemic circulation when orally administered, suffering from their physicochemical properties (size, charge, hydrophilicity) and sensitivity to degradation by gastric and intestinal proteases [48]. However, the intestinal absorption of these biologicals to the circulation, particularly those of the IgG isotype (such as bevacizumab), is possible by means of the neonatal Fc receptor (FcRn) expressed in the intestinal villous enterocytes [49]. FcRn mediates bidirectional transport and immune response to IgG and IgG immune complexes in the gut [50]. In primates, including humans, the expression of FcRn in the enterocytes has been confirmed throughout adult life [51]. On the contrary, in the intestinal epithelium of rodents, FcRn expression would be only limited to the suckling period [52], being responsible for 80% of IgG uptake from the duodenum [49]. This lack of FcRn in adult rats could explain the very low oral bioavailability value obtained with B-NP-P, in which bevacizumab was encapsulated in the free form, compared to B-DS-NP-P, in which the monoclonal antibody was encapsulated forming a complex with sodium deoxycholate. In any case, it is evident that the presence of sodium deoxycholate promotes the oral absorption of bevacizumab. In principle, the effect of sodium deoxycholate may be explained at least by a triple mechanism. First, it has been described that sodium deoxycholate can transiently disrupt the integrity of the tight junctions between intestinal epithelial cells [53]. As a result, the paracellular pathway would be open and the passage of a macromolecule such as bevacizumab facilitated. This mechanism was partially confirmed by the evaluation of the intestinal disruption effect caused by DS and DOCU in a transgenic *C. elegans* model (Fig. 4).

Second, sodium deoxycholate is an ionic detergent capable of disrupting cell membranes and protein to protein interactions [54, 55] and, thus, may increase the fluidity of cell membranes in the gastrointestinal tract. Moreover, it has been described that fluidifying agents (i.e., detergents) can fractionate the plasma membrane in vivo, and vesicles formed would connect immediately to physiological membrane-trafficking mechanisms [3, 56]. In consequence, this increased fluidity may facilitate the absorption of bevacizumab via a pinocytosis process, involving the uptake of fluid and molecules by the cells lining the intestine.

Third, sodium deoxycholate can also act as protease inhibitors to enhance oral absorption. In fact, bile salts have been shown to inhibit brush border membrane and cytosolic proteolytic hydrolysis and would thus be useful to reduce intestinal degradation of peptide drugs [57]. Thus, a macromolecule such as bevacizumab, once released from the nanoparticle, could maintain its integrity for a longer period and facilitate its intact absorption by the transcellular route and/or pinocytosis.

## Conclusions

In summary, the study demonstrates the successful encapsulation of bevacizumab in PEG-coated albumin nanoparticles, particularly with DS as the counterion, leading to improved oral bioavailability. Albumin nanoparticles (containing either DS or DOCU) were successfully prepared by desolvation and then coated with PEG 35,000 to confer stability and mucus-permeating properties. In *C. elegans* FT63, DS and DOCU, either free or encapsulated, disrupted the integrity of the intestinal epithelium, without affecting the overall survival of the worms. The relative oral bioavailability of bevacizumab was calculated to be up to 3.7%. For comparative purposes, this value is higher than the reported oral bioavailability of semaglutide (Rybelsus®, about 1%) [58] or octreotide (Mycapssa®, about 0.7%) [59]. The presence of sodium deoxycholate appears to play a critical role in enhancing the oral absorption of bevacizumab through various potential mechanisms, including tight junction disruption, increased fluidity of membranes, and/or enzymatic inhibition.

### Supplementary Information

Below is the link to the electronic supplementary material.

Supplementary file1 (DOCX 237 KB)

## Data Availability

The datasets generated during and/or analyzed during the current study are available from the corresponding author on reasonable request.
