# Supplementary material for: Mucus-penetrating and permeation enhancer albumin-based nanoparticles for oral delivery of macromolecules: Application to bevacizumab
Source: Drug Deliv Transl Res. 2023 Oct 26;14(5):1189–205. doi: 10.1007/s13346-023-01454-0 (PMC10984897; doi:10.1007/s13346-023-01454-0)
Supplement: Supplementary file 1 — Supplementary file1 (DOCX 237 KB) [file 13346_2023_1454_MOESM1_ESM.docx]

**Supplementary Information**

**Title:** Mucus-penetrating and permeation enhancer albumin-based nanoparticles for oral delivery of macromolecules: application to bevacizumab

**Journal:** Drug Delivery and Translational Research

**Author names:** C. Pangua, S. Espuelas, C. Martinez-Oharriz, J.L. Vizmanos, J.M. Irache

**Corresponding author:** Prof. Juan M. Irache

Dept. Chemistry and Pharmaceutical Technology University of Navarra. C/ Irunlarrea, 1. 31008 – Pamplona, Spain.

E-mail: jmirache@unav.es

**HIP complex dissociation**

The dissociation of HIP complexes was evaluated by incubating samples with 4 mg bevacizumab, under magnetic stirring at 300 rpm, in 5 mL of an aqueous medium (water, gastric simulated fluid at pH 1.6, or intestinal simulated fluid at pH 6.8). After 30 min of incubation, samples were taken and centrifuged at 21,000 x g for 15 min at 4 °C (Sigma 3K30 Osterodeam Harz, Germany). The amount of bevacizumab in the supernatants was quantitated by HPLC and the percentage of dissociated bevacizumab was calculated as the quotient between the amounts of unbound and initially added bevacizumab. The dissociation was expressed in percentage. These results are shown in Figure 1S.

**
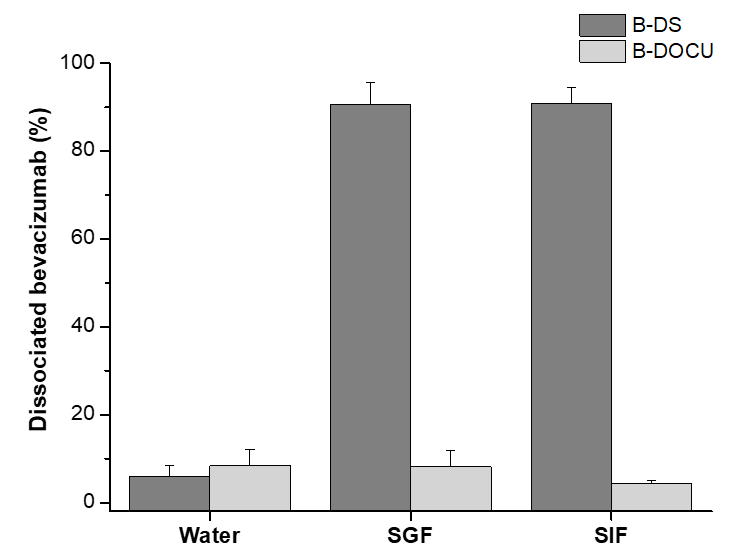
**

Figure 1S. HIP complex dissociation in water, simulated gastric (SGF) and simulated intestinal fluids (SIF). Data expressed as mean ± SD, *n* = 3. Bevacizumab was quantified by HPLC.

***Ex vivo* mucus diffusion in pig intestinal mucus**

Four mg of Lumogen® Red-labelled nanoparticles (4 mg/mL) were dispersed in 0.5 g pig intestinal mucus. Samples were incubated for 2h at 37 °C at 60 rpm (Labnet VorTemp 56 EVC, Labnet International, Inc., Edison NJ). The movement of nanoparticles were recorded in two-dimensional plane at 30 frames/s during 10 s by a high-speed camera (Allied Vision Technologies, Stadtroda, Germany) attached to a wide-field epifluorescence microscope used at 63x magnification oil immersion lens (Leica DM IRB, Wetzlar, Germany). A minimum of 100 trajectories were captured and later tracked and analyzed using an image processing software (Fiji ImageJ).

The diffusion coefficient of the nanoparticles in water (D°) was obtained from the Stokes-Einstein equation [28], whereas the “Effective Diffusion Coefficient” (<Deff>) was calculated as follows:

| $\text{Deff}\text{ =}\frac{\text{ <MSD>}}{\text{4· }\text{Δ}\text{t}}$ |  |
| --- | --- |

in which <MSD> is the mean square displacement of 100 individual trajectories, 4 is a constant related to the 2-dimensional mode of video capture and Δt is the selected time interval. All the formulations were expressed as the ratio (%) between their Deff and their D° (diffusions in mucus and in water, respectively).

Table S1 summarizes the diffusion parameters of the nanoparticles in pig intestinal mucus.

**Table S1.** Diffusion of nanoparticles in pig intestinal mucus obtained by the MPT technique. <Deff>: Effective Diffusion Coefficient; D° (water): diffusion coefficient in water; <Deff>/D°: quotient between the diffusion coefficients of nanoparticles in mucus and water (expressed in percentage); R: ratio between <Deff>/D° of the tested formulations and the value of the control group (NP-P). Data are expressed as mean ± SD (*n* = 3). **: *p* < 0.001 compared to NP-P.

|  | **<Deff> 10^−9^**  **(cm^2^ · S^−1^)** | **D° 10^−9^**  **(cm^2^ · S^−1^)** | **<Deff>/D°**  **(%)** | **R** |
| --- | --- | --- | --- | --- |
| **NP-P** | 0.243 ± 0.134 | 29.960 | 0.812 ± 0.189 | 1 ± 0.153 |
| **DOCU-NP-P** | 0.128 ± 0.066 | 22.358 | 0.570 ± 0.105 | 0.702 ± 0.085 |
| **DS-NP** | 0.075 ± 0.043 | 28.088 | 0.257 ± 0.072 | 0.316 ± 0.058 |
| **DS-NP-P** | 0.305 ± 0.034 | 24.747 | 1.221 ± 0.038** | 1.503 ± 0.031 |

**Intake evaluation study in *C. elegans***

*C. elegans* transgenic strain FT63 labelled with green fluorescent protein (GFP) at the epithelial junctions (DLG::GFP) was obtained from the Caenorhabditis Genetics Centre (CGC, University of Minnesota, MN, USA). To evaluate the ingestion of nanoparticles by the worms, L4 larvae worms were cultured in NGM plates supplemented with Lumogen® Red-loaded nanoparticles for two hours. After that, worms were collected with PBST (0.01% Triton X-100 in Phosphate Buffered Saline) and placed in a 2% agarose pad with 1% of sodium azide (w/v). Samples were visualized using an Automated Microscope Zeiss Axio Imager M1 with an Axiocam MRm camera (Zeiss Microscopy, Jena, Germany), using the rhodamine filter to visualize the Lumogen® Red-loaded nanoparticles and the GFP filter to visualize the GFP fluorescence of the transgenic worms. The images were taken with ZEN software (Zeiss Microscopy, Jena, Germany) and processed with ImageJ. Figure 2S shows the intake of nanoparticles by worms.


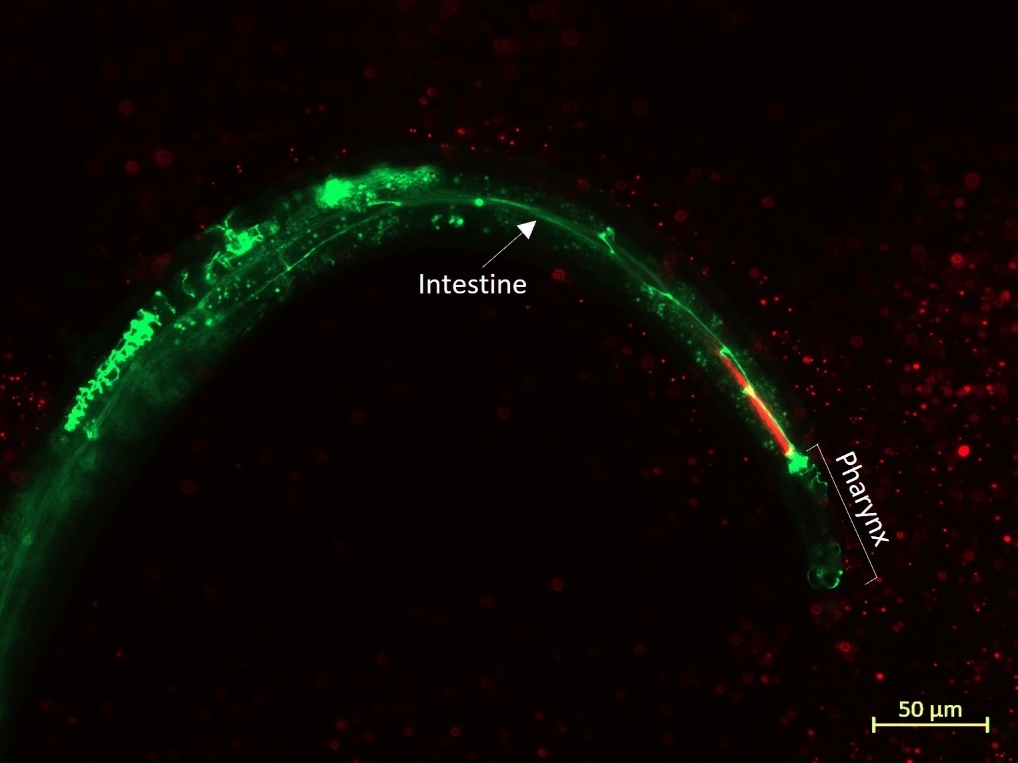


Figure 2S. Fluorescent image of a FT63 *C. elegans* worm growth in NGM supplemented with Lumogen Red-loaded nanoparticles (NP-P). The epithelial junctions of the transgenic worm, labelled with GFP, appear in green.

**Life span assay in *C. elegans***

The lifespan assay was conducted in L4 larvae stage seeded in NGM plates. Dead worms were counted and removed every 2 days until day 15 when the counted was daily until the end of the study. Nematodes were considered dead if they did not move after repeated mechanical stimuli. Figure 3 S shows the Kaplan-Meier representations of the percentage of worms alive over time for the different treatments tested.


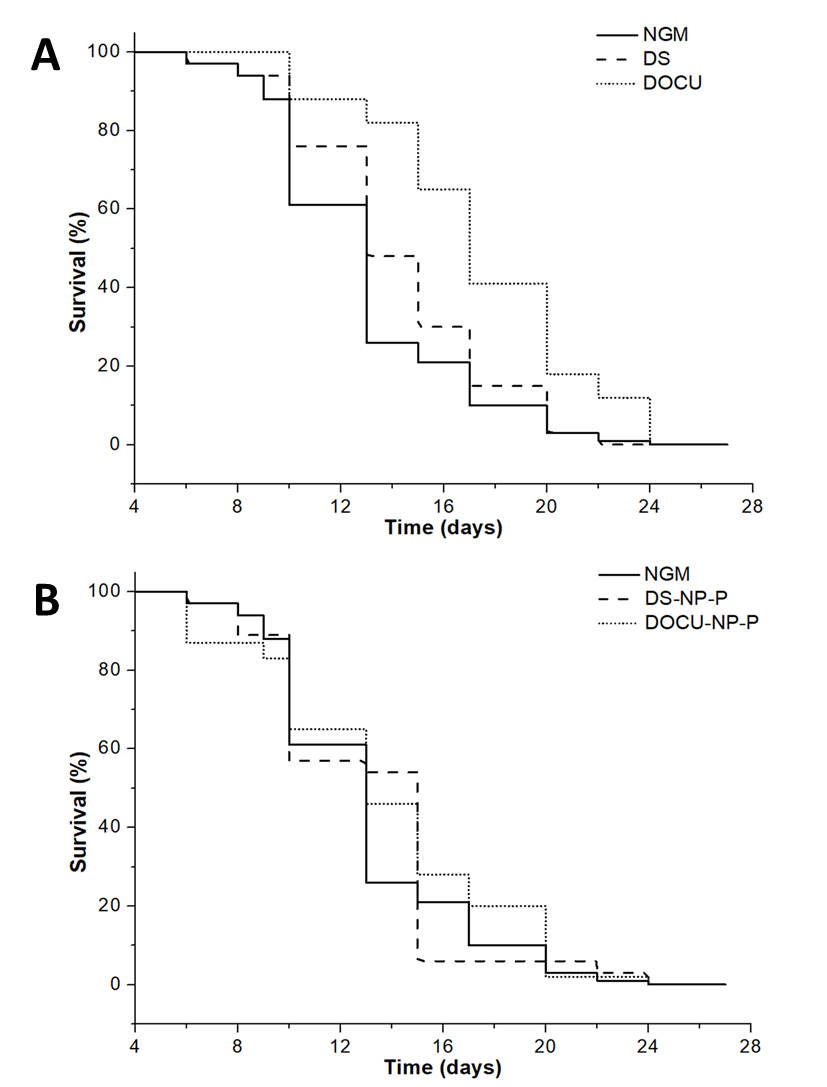


Figure 3S. Effect of nanoparticles on the lifespan of *C. elegans*. Kaplan-Meier representation of the percentage of worms alive over time (*n* ≥ 70 worms). (A) NGM: control; DS: free sodium deoxycholate, DOCU: free sodium docusate. (B) NGM: control; DS-NP-P: DS encapsulated into PEG-coated albumin nanoparticles, DOCU-NP-P: DOCU encapsulated into PEG-coated albumin nanoparticles.
